# Low-Dose Metronomic Topotecan and Pazopanib (TOPAZ) in Children with Relapsed or Refractory Solid Tumors: A C17 Canadian Phase I Clinical Trial

**DOI:** 10.3390/cancers14122985

**Published:** 2022-06-17

**Authors:** Arif Manji, Yvan Samson, Rebecca J. Deyell, Donna L. Johnston, Victor A. Lewis, Alexandra P. Zorzi, Jason N. Berman, Kathy Brodeur-Robb, Ellen Morrison, Lynn Kee, Sushil Kumar, Sylvain Baruchel, James A. Whitlock, Daniel A. Morgenstern

**Affiliations:** 1 The Hospital for Sick Children, University of Toronto, Toronto, ON M5G 1X8, Canada; arif.manji@sickkids.ca (A.M.); lynn.kee@sickkids.ca (L.K.); sylvain.baruchel@sickkids.ca (S.B.); jim.whitlock@sickkids.ca (J.A.W.); 2 CHU Sainte-Justine, Montréal, QC H3T 1C5, Canada; yvan.samson@umontreal.ca; 3 BC Children’s Hospital, Vancouver, BC V6H 3N1, Canada; rdeyell@cw.bc.ca; 4 Children’s Hospital of Eastern Ontario, Ottawa, ON K1H 8L1, Canada; djohnston@cheo.on.ca (D.L.J.); jberman@cheo.on.ca (J.N.B.); 5 Alberta Children’s Hospital, Calgary, AB T3B 6A8, Canada; victor.lewis@albertahealthservices.ca; 6 Children’s Hospital, London Health Sciences Centre, London, ON N6A 5W9, Canada; alexandra.zorzi@lhsc.on.ca; 7 C17 Council for Children’s Cancer and Blood Disorders, Edmonton, AB T6G 2E1, Canada; kathy.brodeur-robb@c17.ca (K.B.-R.); ellen.morrison@c17.ca (E.M.); 8 Drug Metabolism & Pharmacokinetics, QPS Holdings LLC, Newark, DE 19711, USA; sushilkumar29@yahoo.ca

**Keywords:** phase I trial, pediatric, children, pazopanib, topotecan, metronomic, angiogenesis

## Abstract

**Simple Summary:**

Low-dose continuous oral chemotherapy may work together with targeted tyrosine kinase inhibitors to target a cancer’s ability to promote new blood supply (angiogenesis). We undertook a phase I study of the combination of oral topotecan and pazopanib in children with relapsed or refractory solid tumors to determine the optimal safe dose. Overall, the treatment combination was well tolerated with few severe side effects. Although there were no objective responses, stable disease was achieved in 40% of treated patients, suggesting that this combination may have a role in the maintenance setting following treatment with alternative chemotherapy regimens.

**Abstract:**

Oral metronomic topotecan represents a novel approach to chemotherapy delivery which, in preclinical models, may work synergistically with pazopanib in targeting angiogenesis. A phase I and pharmacokinetic (PK) study of this combination was performed in children with relapsed/refractory solid tumors. Oral topotecan and pazopanib were each administered daily without interruption in 28-day cycles at five dose levels (0.12 to 0.3 mg/m^2^ topotecan and 125 to 160 mg/m^2^ pazopanib powder for oral suspension (PfOS)), with dose escalation in accordance with the rolling-six design. PK studies were performed on day 1 and at steady state. Thirty patients were enrolled, with 26 evaluable for dose-limiting toxicity (DLT), with median age 12 years (3–20). Toxicities were generally mild; the most common grade 3/4 adverse events related to protocol therapy were neutropenia (18%), thrombocytopenia (11%), lymphopenia (11%), AST elevation (11%), and lipase elevation (11%). Only two cycle 1 DLTs were observed on study, both at the 0.3/160 mg/m^2^ dose level comprising persistent grade 3 thrombocytopenia and grade 3 ALT elevation. No AEs experienced beyond cycle 1 required treatment discontinuation. The best response was stable disease in 10/25 patients (40%) for a median duration of 6.4 (1.7–45.1) months. The combination of oral metronomic topotecan and pazopanib is safe and tolerable in pediatric patients with solid tumors, with a recommended phase 2 dose of 0.22 mg/m^2^ topotecan and 160 mg/m^2^ pazopanib. No objective responses were observed in this heavily pre-treated patient population, although 40% did achieve stable disease for a median of 6 months. While this combination is likely of limited benefit for relapsed disease, it may play a role in the maintenance setting.

## 1. Introduction

Maintenance chemotherapy has played a significant role in improving survival from pediatric acute lymphoblastic leukemia [1]. Experience with maintenance therapy in pediatric solid tumors, however, has been largely limited to immunotherapy and retinoic acid in neuroblastoma [2], and vinorelbine and cyclophosphamide in rhabdomyosarcoma [3]. An oral maintenance regimen would be of interest for children with solid tumors, with the goal of maintaining remission following multimodal therapy while minimizing toxicity and supporting quality of life.

Metronomic drug administration represents a novel approach to maintenance chemotherapy delivery, involving frequent or continuous administration of cytotoxic agents at low doses without prolonged drug-free intervals [4]. Metronomic therapy has been associated with tumor response in animal models, even in the setting of acquired drug resistance to the same agents administered conventionally [5], through mechanisms of action that include anti-angiogenesis [6]. For example, the MEMMAT regimen (incorporating bevacizumab with continuous oral celecoxib, thalidomide, and fenofibrate, together with low-dose cyclophosphamide/etoposide) is currently being evaluated for pediatric brain tumors [7,8]. Accordingly, there has been interest in the potential synergy between metronomic chemotherapy and tyrosine kinase inhibitors (TKI) with potent anti-angiogenic properties [9,10].

Pazopanib is a multitargeted tyrosine kinase inhibitor of vascular endothelial growth factor (VEGF) receptors 1, 2, and 3; platelet-derived growth factor receptors; and c-Kit pathways and is approved by the US Food and Drug Administration for use in adults with advanced renal cell carcinoma and soft tissue sarcomas [11,12]. A Children’s Oncology Group (COG) phase I study of pazopanib in children with recurrent or refractory solid tumors [13] established the single-agent maximum tolerated dose (MTD) to be 450 mg/m^2^/dose for tablets and 160 mg/m^2^/dose for the powder for oral suspension (PfOS). A phase II randomized trial of neoadjuvant chemoradiotherapy with or without pazopanib in pediatric and adult soft tissue sarcoma [14] was terminated early because of a significant improvement in pathological response rate in the pazopanib arm (58 vs. 22%), although further outcome data are not yet available.

Preclinical studies have demonstrated potential synergy between pazopanib and metronomic topotecan in models of adult malignancies [9,10,15,16,17,18], and in pediatric models of neuroblastoma, osteosarcoma, and rhabdomyosarcoma [19,20]. Pazopanib in combination with intermittent dosing of oral topotecan has been evaluated in adults, both in a phase I trial of patients with advanced solid tumors [21] and in a phase II trial of patients with advanced sarcoma [22]. However, this dosing approach with weekly oral topotecan at higher doses was associated with grade 3 or 4 toxicity in most patients, with rates of discontinuation and dose interruption due to adverse events of 29% and 38%, respectively. A phase I trial of oral metronomic topotecan and pazopanib in adults with recurrent or persistent gynecologic tumors [23] identified a recommended phase 2 dose (RP2D) of topotecan 0.25 mg and pazopanib 600 mg once daily, with an overall response rate of 28%.

This multicenter phase I clinical trial was performed to determine the maximum-tolerated dose (MTD) of oral metronomic topotecan and pazopanib PfOS in combination, to define the associated toxicities, and to characterize relevant pharmacokinetic (PK) variables in children with relapsed or refractory solid tumors.

## 2. Materials and Methods

### 2.1. Patient Eligibility

This study was conducted by the C17 Council for Children’s Cancer and Blood Disorders with 10 participating sites across Canada. Patients aged 2 to 21 years with relapsed or refractory solid tumors were eligible. Patients with primary CNS tumors were eligible for enrolment at dose levels 0–2 but excluded from enrolment at dose levels 3–5 due to escalation of the pazopanib dose and reports of tumor-associated hemorrhage in other studies of pazopanib at higher doses [13]. A histologic verification of malignancy was required, except in patients with optic pathway glioma or pineal tumors with tumor marker elevation. Other eligibility criteria included recovery from acute effects of prior therapy; performance status (Lansky/Karnofsky) of ≥50; adequate renal, hepatic, cardiac, CNS, and hematologic function; and normal blood pressure on stable doses of no more than one anti-hypertensive. Exclusion criteria included known CNS metastasis; active bleeding; history of thromboembolic events; active anticoagulation or antiplatelet agents; uncontrolled infection; recent or planned major surgical procedure; pregnancy or lactation; or concurrent use of CYP3A4/PgP substrates, QTc prolonging medications, or other anticancer agents.

Research ethics board approval was obtained at all participating sites, and written informed consent and assent were obtained in accordance with federal and institutional guidelines.

### 2.2. Drug Administration

Pazopanib was supplied by Novartis Pharmaceuticals Canada Inc. (Dorval, QC, Canada). Topotecan was obtained commercially. Both topotecan and pazopanib were administered orally once daily without interruption on an empty stomach in 28-day cycles, except for cycle 1, day 1 on which day pazopanib was not administered to facilitate topotecan PK. Three dose levels were initially planned, with further dose escalation incorporated following amendment, with a starting dose of 0.12 mg/m^2^ and 125 mg/m^2^ of topotecan and pazopanib PfOS, respectively. Interpatient dose escalation was planned according to the Rolling Six design [24], with additional dose levels of 0.16 and 125 mg/m^2^, 0.22 and 125 mg/m^2^, 0.22 and 160 mg/m^2^, 0.3 and 160 mg/m^2^, and 0.4 and 160 mg/m^2^ of topotecan and pazopanib PfOS, respectively.

### 2.3. Study Evaluations

History, physical examination, blood-pressure monitoring, and laboratory studies were obtained routinely. Cardiac assessment including ECG and echocardiography, and X-ray imaging of the tibial growth plates occurred at baseline, before cycles 2 and 5, and then at every sixth cycle. Adverse events were graded according to the National Cancer Institute Common Terminology Criteria for Adverse Events (CTCAE), version 4. Disease evaluations were obtained at baseline, prior to cycles 3 and 5, and then after every third cycle. Response was evaluated using RECIST 1.1 [25] with an MIBG response evaluation by the Curie score for MIBG-positive lesions in neuroblastoma [26].

### 2.4. Pharmacokinetics

EDTA blood samples (2 mL) were obtained at each timepoint. Plasma was obtained by centrifugation and stored locally at −80 °C before batch shipping for central analysis. The topotecan PK analysis incorporated a PK profile on cycle 1, day 1 (C1D1) prior to initiation of pazopanib, with timepoints at baseline; 15 and 30 min; and 1, 3, 6, 8, and 24 h post-dose. A similar profile was obtained on C2D1 with concurrent pazopanib administration. Trough topotecan levels were obtained on C1D15 and D1 of every odd numbered cycle. Pazopanib trough PK samples were obtained on C1D15, C2D1, and D1 of every odd-numbered cycle. Concentrations of topotecan and pazopanib were determined using high-performance liquid chromatography tandem mass spectrometry performed by the Analytical Facility for Bioactive Molecules, Hospital for Sick Children, Toronto, Canada. Non-compartmental analysis of topotecan PK profiles was performed using PKSolver 2.0 [27], with AUC calculated using the linear trapezoidal method.

## 3. Results

### 3.1. Patient Characteristics

From March 2015 to April 2019, 30 patients were enrolled. The patient characteristics are summarized in Table 1. All patients enrolled had received at least one prior therapy including chemotherapy, radiotherapy, or both.

### 3.2. Toxicities

Four patients were not evaluable for dose-limiting toxicity (DLT) (early disease progression, *n* = 3; early withdrawal of consent, *n* = 1). The dose escalation schema and summary of DLTs are presented in Table 2. Toxicities attributable to topotecan or pazopanib during all cycles are listed in Table 3. The most common grade 3 or 4 adverse events (AEs) related to protocol therapy in cycle 1 were neutropenia (18%), thrombocytopenia (11%), lymphopenia (11%), aspartate aminotransferase (AST) elevation (11%), and lipase elevation (11%). Two cycle 1 DLTs were observed upon study, both at dose level 4, comprising persistent grade 3 thrombocytopenia and grade 3 alanine aminotransferase (ALT) elevation. Two of five patients at dose level 4, and zero of six patients at the prior dose level experienced DLT; thus, the recommended phase 2 dose was determined to be dose level 3 (topotecan 0.22 mg/m^2^/day and pazopanib PfOS 160 mg/m^2^/day). No AEs experienced beyond cycle 1 required treatment discontinuation.

### 3.3. PK Studies

The initial planned dose levels (0–2) used a fixed dose of pazopanib of 125 mg/m^2^/day while escalating topotecan. The overall mean trough concentration (C_trough_) of pazopanib at C1D15 was 20.1 µg/mL, consistent with those previously reported for single agent pazopanib suspension in paediatric patients [13] and equivalent to the threshold for clinical activity established in renal cell carcinoma [28]. However, due to interpatient variability, only 7 of the 14 patients (50%) achieved C_trough_ > 20 µg/mL and the protocol was therefore amended to test 160 mg/m^2^/day pazopanib, equivalent to the single-agent pediatric RP2D for PfoS formulation, at dose levels 3–5 [13]. This dose increase corresponded to a non-statistically significant increased mean C_trough_ of 33.8 µg/mL (*p* = 0.06), with 7 of 11 patients (64%) achieving C_trough_ > 20 µg/mL. Overall mean C_trough_ pazopanib in all patients was 26.1 µg/mL at C1D15, and mean C_trough_ was > 20 µg/mL at all subsequent timepoints. Single-factor ANOVA demonstrated that topotecan dose levels (0.12, 0.16, 0.22, and 0.3 mg/m^2^) did not impact corresponding dose-normalized pazopanib C_trough_ at C1D15 (*p* = 0.39; F = 1.05). In addition, there was no statistically significant difference (*p* = 0.32) in pazopanib C_trough_ at C1D15 between 120 mg/m^2^ (mean 0.161 ng/mL per mg/m^2^) and 160 mg/m^2^ (mean: 0.211 ng/mL per mg/m^2^), which indicates dose-proportionality of pazopanib. The mean drug accumulation ratio calculated from C_trough_ values of paired samples at C1D15 and C2D1 is 1.21 ± 0.76.

**Figure 1:**
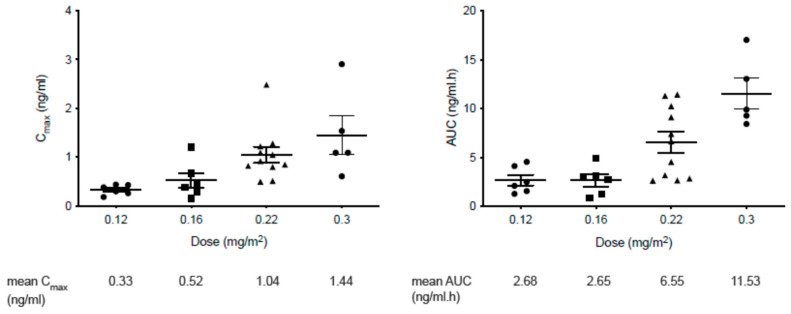
Topotecan mean peak serum concentration (C_max_) and area under the curve (AUC) by dose level (as indicated by different shapes for data points within each dose level).

Topotecan doses were escalated from 0.12 to 0.3 mg/m^2^/day, with PK analysis demonstrating a corresponding increase in mean C_max_ and AUC_0–∞_ (Figure 1). There was no difference in the PK profile of topotecan for C1D1 (prior to pazopanib) compared to C2D1 (on pazopanib) in the eight patients for whom paired data were available (*p* = 0.11 by paired t-test for comparison of AUC_0–∞_). An analysis of the serial trough topotecan concentrations in subsequent cycles did not show any trend of drug accumulation (data not shown). Linear regression shows a clear linear relationship between dose and C_max_ (r^2^ = 0.9325) and also between dose and AUC_0–∞_ (r^2^ = 0.8262) of topotecan. C_max_ for dose of 0.3 mg/m^2^ is significantly higher than that for 0.12 mg/m^2^ (*t*-test, *p* = 0.047). AUC_0–∞_ for dose of 0.3 mg/m^2^ is significantly higher than that for 0.12 mg/m^2^ (*t*-test, *p* = 0.01).

### 3.4. Response Evaluation

**Figure 2:**
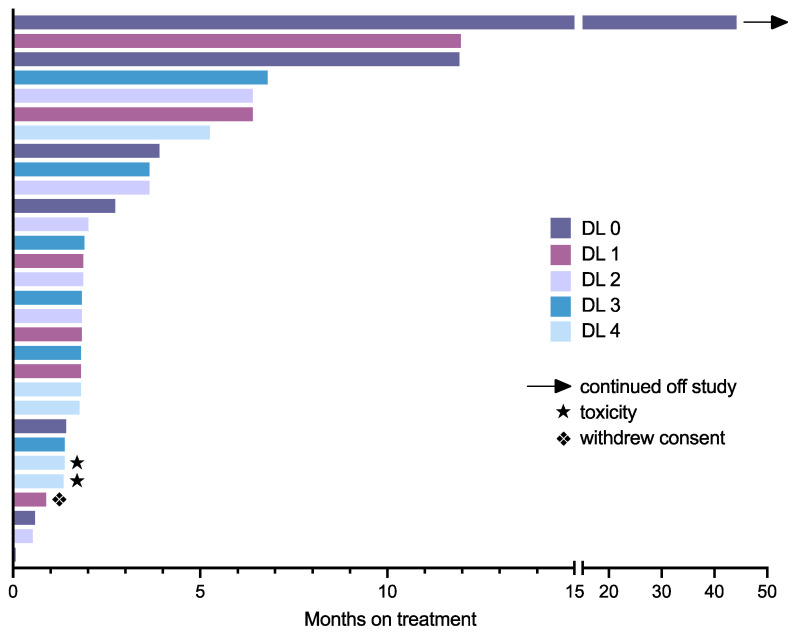
Swimmer’s plot for patients in the study. Except where indicated, all patients discontinued therapy due to progressive disease. Dose level (DL) for each patient as indicated.

Patients remained on study therapy for a median duration of 1.9 months (0.1–44.2), as presented in Figure 2. Of these, 25 patients were evaluable for response, having undergone at least one disease evaluation following the start of therapy. Of the five patients deemed inevaluable for response, three discontinued therapy prior to evaluation due to toxicity (2) or withdrawal of consent (1), with the remaining two having clinical progression of disease without formal disease re-evaluation. The best response was stable disease in 10 patients (40%) with the following tumors: neuroblastoma (4), osteosarcoma (3), Ewing sarcoma/PNET (2), and medulloblastoma (1). The median duration of stable disease was 6.4 months (1.7–45.1). One patient with refractory neuroblastoma achieved stable disease for 45 months and continued on topotecan and pazopanib via compassionate access after study closure. This patient had MIBG-avid bone disease confirmed to be metabolically active on the FDG-PET scan. They were treated on dose level 0 and, as anticipated, had relatively lower PK values with cycle 1 topotecan C_max_ 0.17 mg/m^2^ and AUC 1.27 g/mL·h; pazopanib C_trough_ 13.3 µg/mL at C1D15, although mean pazopanib C_trough_ was 20.6 µg/mL through cycles 2–47.

## 4. Discussion

In this study, the combination of oral metronomic topotecan and pazopanib administered daily to pediatric patients with recurrent or refractory solid tumors was well tolerated overall with grade 3 thrombocytopenia and ALT elevation as the only DLTs. The most common toxicities were hematologic, including neutropenia, thrombocytopenia, and anemia. The MTD and RP2D of this regimen is topotecan 0.22 mg/m^2^ and pazopanib PfOS 160 mg/m^2^ both on a once daily schedule. The possibility of additional late effects from prolonged use of maintenance therapies (such as topotecan/pazopanib) needs to be considered, particularly in the pediatric population. At present, there are no published data in this regard and long-term follow-up was not possible in the context of this phase I study.

Data from adult trials of pazopanib in advanced solid tumors and renal cell carcinoma suggest that a steady state concentration of 20.5 µg/mL is associated with improved efficacy. In this study, 64% (7/11) patients at the RP2D of pazopanib exceeded this concentration at C1D15. Of note, the patient with the longest stable disease on study had a relatively low C1D15 C_trough_ (13.3 µg/mL), although they had a mean C_trough_ of 20.6 µg/mL across the remaining cycles on therapy. Combination therapy did not seem to have a significant impact on the topotecan PK profile, nor was there any evidence of topotecan accumulation in later cycles. Pharmacokinetic data reveal that the dose level of topotecan does not impact trough concentrations of pazopanib. In the present study, the absence of differences in PK profiles of topotecan between C1D1 (topotecan administered alone) and C2D1 (topotecan co-administered with pazopanib) indicates that pazopanib at 120 or 160 mg/m^2^ does not impact topotecan pharmacokinetics. There are conflicting reports regarding the effect of pazopanib on topotecan pharmacokinetics. A pharmacokinetic study conducted in gynecological cancer patients [29] did not reveal any impact of pazopanib at 400, 600, or 800 mg on topotecan pharmacokinetics (administered 0.25 mg daily), which was consistent with pre-clinical findings [19]. On the other hand, another study showed that 800 mg pazopanib administered in tablet form caused 1.7-fold increase in the exposure of topotecan at doses of 8 mg weekly or 2.5 mg daily (five days) [21]. However, in this study, the dose of topotecan (>1.42 mg/m^2^) was 10-fold higher compared to the studies where low-dose metronomic topotecan was administered daily.

While there were no objective responses observed in this heavily pretreated patient population, a significant proportion (40%) of patients achieved stable disease for a median duration of 6.4 months, with one patient continuing to have stable disease for 45 months and remaining on therapy via compassionate access after the study closure. Adult phase 3 data of pazopanib in metastatic soft tissue sarcoma also failed to show a significant objective response rate (6%) or improvement in overall survival; rather, it was the statistically significant improvement in progression-free survival (PFS) that led to regulatory approval [12]. The results of several phase II trials of receptor tyrosine kinase inhibitors in the treatment of bone sarcomas also suggest that objective response rate in the setting of relapsed or refractory disease may be of less relevance in evaluating their efficacy, raising the potential for their role as maintenance therapy [30].

## 5. Conclusions

Based on the findings from this study, the combination of oral metronomic topotecan and pazopanib appears to be a safe and tolerable regimen at the RP2D identified; the lack of objective responses suggests that this combination is likely of limited benefit for relapsed disease but may play a role as maintenance therapy, perhaps in high-risk neuroblastoma or soft-tissue sarcoma.

## Figures and Tables

**Table 1:** Characteristics of eligible patients.

Characteristic	Value
Age, years	
Median	12
Range	3–20
Sex	
Male	13
Female	12
Diagnosis	
Sarcoma	
Osteosarcoma	8
Rhabdomyosarcoma	4
Ewing Sarcoma/PNET	4
Synovial sarcoma	1
Neuroblastoma	7
Brain Tumor	
Ependymoma	2
High-grade glioma	1
Medulloblastoma	1
Adrenocortical carcinoma	1
Wilms Tumor	1
Prior chemotherapy regimens, number	
Median	1.5
Range	0–10
Prior radiation therapy	22

**Table 2:** Summary of dose levels and dose-limiting toxicities in cycle 1.

Dose Level	Topotecan Dose (mg/m^2^/Day)	Pazopanib Dose (mg/m^2^/Day)	Patients Entered	Patients Evaluable	Patients with DLT
0	0.12	125	6	5	0
1	0.16	125	6	5	0
2	0.22	125	7	5	0
3	0.22	160	6	6	0
4	0.3	160	5	5	2 *

* DLTs: persistent grade 3 thrombocytopenia (*n* = 1) and persistent grade 3 ALT elevation (*n* = 1).

**Table 3:** Summary of treated-related toxicities.

	Number of Patients			
	Maximum Grade Observed per Patient during Cycle 1 (*n* = 28)		Maximum Grade Observed per Patient across All Cycles (*n* = 28)	
Toxicity Type	Any Grade	Grade 3/4	Any Grade	Grade 3/4
				
**Hematologic**				
Thrombocytopenia	12	3	14	4 *
Neutropenia	15	5	19	9
Lymphopenia	13	3	20	4
Anemia	16	2	20	5
				
**Non-hematologic †**				
GI/metabolic				
Constipation	7	0	10	0
Diarrhea	3	0	8	1
Nausea	11	0	14	0
Vomiting	9	0	13	0
Anorexia	8	0	15	0
ALT elevation	12	2	14	2 *
AST elevation	13	2	18	3
Bilirubin elevation	5	0	8	0
Hypoalbuminemia	6	0	10	1
Lipase elevation	4	2	7	3
Creatinine elevation	3	0	4	0
Hypocalcemia	4	0	12	1
Hyperkalemia	3	0	9	0
Hypokalemia	3	0	3	0
Hypomagnesemia	4	0	7	0
Hypophosphatemia	5	0	8	0
Cardiac/respiratory				
Sinus tachycardia	3	0	4	0
Cough	5	0	9	0
Nervous system				
Ataxia	3	0	3	0
Headache	4	0	6	1
Psychiatric				
Anxiety	3	0	5	0
Insomnia	3	0	5	0
Musculoskeletal/constitutional				
Fatigue	11	0	17	0
Pain	6	0	13	1

* Dose-limiting toxicity. † Non-hematologic toxicities related to protocol therapy that occurred in >10% of patients during the first cycle of protocol therapy.

## Data Availability

The data presented in this study are available on request from the corresponding author.
